# Nedd4-2 haploinsufficiency causes hyperactivity and increased sensitivity to inflammatory stimuli

**DOI:** 10.1038/srep32957

**Published:** 2016-09-08

**Authors:** Sudhirkumar Yanpallewar, Ting Wang, Dawn C. I. Koh, Eros Quarta, Gianluca Fulgenzi, Lino Tessarollo

**Affiliations:** 1 Neural Development Section, Mouse Cancer Genetics Program, CCR, NCI, Frederick, MD, 21702, USA; 2 Physiological Science Section, Department of Experimental and Clinical Medicine, University of Florence, Italy; 3 Department of Molecular and Clinical Sciences, Marche Polytechnic University, Ancona, Italy

## Abstract

Nedd4-2 (NEDD4L in humans) is a ubiquitin protein ligase best known for its role in regulating ion channel internalization and turnover. Nedd4-2 deletion in mice causes perinatal lethality associated with increased epithelial sodium channel (ENaC) expression in lung and kidney. Abundant data suggest that Nedd4-2 plays a role in neuronal functions and may be linked to epilepsy and dyslexia in humans. We used a mouse model of Nedd4-2 haploinsufficiency to investigate whether an alteration in Nedd4-2 levels of expression affects general nervous system functions. We found that Nedd4-2 heterozygous mice are hyperactive, have increased basal synaptic transmission and have enhanced sensitivity to inflammatory pain. Thus, Nedd4-2 heterozygous mice provide a new genetic model to study inflammatory pain. These data also suggest that in human, SNPs affecting NEDD4L levels may be involved in the development of neuropsychological deficits and peripheral neuropathies and may help unveil the genetic basis of comorbidities.

Nedd4-2 (neuronal precursor cell expressed developmentally downregulated-4 type 2) belongs to the Nedd4 family of E3 ubiquitin ligases. Its substrates include a wide range of proteins such as epithelial sodium channels (ENaC), Na+-Cl- cotransporter (NCC), chloride and potassium channels, voltage gated sodium (NaV) channels, serum glucocorticoid kinase (sgk), glutamate transporter, and the neurotrophin receptor TrkA [reviewed in ref. 1]. Through these interactions Nedd4-2 has the potential to regulate various aspects of respiratory, cardiovascular, renal and neuronal functions^1^,^2^. Recent studies employing knockout mouse models have uncovered some *in vivo* roles of Nedd4-2 (human Nedd4L) and have validated its role in the regulation of ENaC for normal lung function^3^,^4^. Mice lacking Nedd4-2 die of respiratory failure but the lethality can be rescued by pulmonary administration of an ENaC inhibitor^4^. Moreover, deletion of Nedd4-2 in adult renal tubules causes a salt-sensitive hypertension with hypercalciuria that can be reversed by a NCC inhibitor, confirming a role for Nedd4-2 in regulating NCC levels^5^,^6^. Nedd4-2 expression in the brain and its potential interaction with a number of neuronal substrates suggest that it may have essential E3 ubiquitin ligase functions in the nervous system. For example, brain deletion of Nedd4-2 leads to increased ENaC expression and hypertension induced by a high-salt diet that can be prevented by central infusion of the ENaC blocker benzamil^7^. However, Nedd4-2 can also regulate glutamate, dopamine and the CHT-1 choline transporters, and interacts with a number of neuronal NaV channels in cortical and dorsal root ganglia (DRG) neurons^8^,^9^,^10^,^11^,^12^. Recently, the identification of a Nedd4L missense mutation in a patient with epileptic encephalopathy and the isolation of missense mutations in highly conserved residues of Nedd4-2 in families with photosensitive generalized epilepsy has suggested that this E3 ligase, in addition to hypertension, is an epilepsy associated gene and can contribute to CNS pathologies^13^. Although specific ablation of Nedd4-2 in mouse models has been critical for the validation of some of its substrates and target organs, to date it is unclear whether partial loss of its activity, that better mimic the human situation due to polymorphisms, can affect mammalian physiology. The finding that Nedd4-2 is haploinsufficient, at least in lung^3^, and another member of the Nedd4 family of E3 ubiquitin ligases, Nedd4-1 is happloinsufficient as well^14^, prompted us to evaluate whether mice with reduced Nedd4-2 levels have specific deficits that may help identify its potential genetic role in human pathologies^3^. We found that Nedd4-2 heterozygous mice have increased locomotor activity and basal synaptic excitability. Moreover, they exhibit a significant increase in inflammatory pain. These data suggest that Nedd4-2 haploinsufficiency in mammals leads to a significant pathological outcome and provides a model to identify pathologies associated with partial Nedd4L loss of function in humans.

## Results

### Nedd4-2 is haploinsufficient

**Figure 1:**
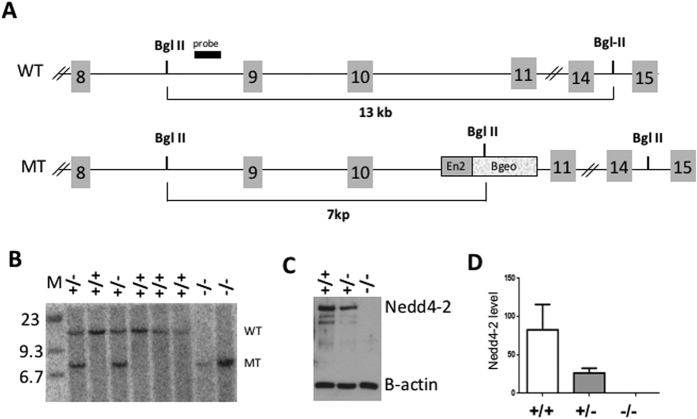
Generation of Nedd4-2 knockout mice. (**A**) Schematic representation of the genetic strategy used to target *Nedd4-2*. The gene-trap cassette composed of the Engrailed 2 (En2) splice-acceptor sequence and the fusion β-galactosidase-neomycin gene (βGeo) is inserted between exons 10 and 11 causing a change in the size of the Bgl II DNA restriction fragment. (**B**) Southern blot using the probe shown in (**A**) and genomic Bgl II-digested DNA from wild-type (+/+), heterozygous (+/−), and homozygous *Nedd4-2* mutant (−/−) mice. (**C**) Western blot analysis of Nedd4-2 protein in lysates from +/+, +/− and −/− mouse E13.5 embryo DRG. Note the loss of about 50% of Nedd4-2 protein in the +/− sample. β-actin was used as a control for loading. (**D**) Quantification of Nedd4-2 band intensity as shown in (**C**) relative to β-actin; n = 3.

**Figure 2:**
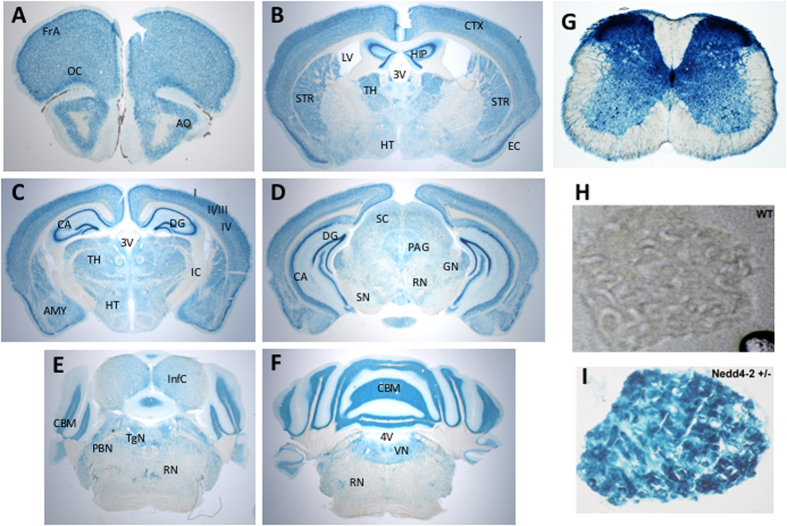
Nedd4-2 is widely expressed in the adult mouse brain. (**A–F**) β-Galactosidase staining of heterozygous mouse brain sections showing widespread expression of NEDD4-2 in the adult brain. High expression in entorhinal cortex cortical layers II and III, striatum, DG and CA regions of hippocampus and cerebellum. FrA- Frontal association cortex, OC-orbital cortex, AO- anterior olfactory nucleus, LV-lateral ventricle, CTX-cortex, HIP-hippocampus, STR-striatum, TH-thalamus, HT-hypothalamus, 3V- third ventricle, EC- entorhinal cortex, IC- internal capsule, AMY- amygdala, SC-superior colliculus, PAG- periaqueductal gray, GN- geniculate nucleus, RN-reticular nucleus, SN-substantia nigra, InfC- inferior colliculus, CBM- cerebellum, TgN- tegmental nucleus, PBN- parabrachial nucleus, 4V- 4^th^ ventricle, VN- vestibular nucleus. (**G**) β-Gal staining of an adult Nedd4-2 heterozygous mouse spinal cord section at the lumbar level showing widespread expression of NEDD4-2 in the gray matter. (**H,I**) Staining of a section from an adult heterozygous mouse lumbar DRG shows Nedd4-2 expression in virtually all DRG neurons (**I**); (**H**) shows an unstained adjacent DRG section.

Nedd4-2 has a role in the regulation of ion channels levels in lung and kidney^3^,^4^,^6^. However, limited information is available on its function in the adult nervous system^2^,^15^. To address this, we targeted the Nedd4-2 gene in mouse by employing an embryonic stem cell line with a gene trap cassette inserted in the intron between exon 10 and 11 (Fig. 1). The cassette used for gene inactivation contains a strong splice acceptor from the Engrail2 gene and the β-geo cassette (fusion between the neo and the β-galactosidase gene) such that, after exon 10 the splicing will occur to the Engrail2 exon and will stop at the β-geo fusion gene preventing splicing to Nedd4-2 exon 11 (Fig. 1A). Importantly, the promoterless β-galactosidase gene driven by the endogenous Nedd4-2 gene allows for the study of Nedd4-2 expression (Fig. 2). Mating of heterozygous mice yielded progeny with WT, Nedd4-2 +/− and Nedd4-2 −/− animals present at the expected ratio. While at birth Nedd4-2 −/− pups were indistinguishable from WT littermates, most of them died early post-natally. This is in agreement with two previous studies reporting perinatal lethality due to lung inflammation and respiratory distress^3^,^4^. Western blot analysis of DRG protein extracts from Nedd4-2 −/− mice showed a complete loss of Nedd4-2 protein suggesting effective and complete inactivation of the gene (Fig. 1C). Importantly, DRG from heterozygous mice showed a reduction in Nedd4-2 protein level of at least 50% suggesting a lack of compensatory mechanisms to keep Nedd4-2 levels constant after loss of one allele. This result is in agreement with the findings of Boase *et al.*^3^ reporting a similar 50% reduction of Nedd4-2 protein level in the lung from heterozygous animals.

Since there is limited information on the spatial expression of Nedd4-2 in the nervous system we employed the promoterless β-galactosidase gene driven by the endogenous Nedd4-2 promoter to investigate Nedd4-2 expression in the brain, spinal cord and DRG neurons. β-Galactosidase staining of serial coronal brain sections revealed a widespread expression of Nedd4-2 in most brain areas with highest levels in the cortex, hippocampus and cerebellum, whereas low levels of expression were present in the substantia nigra and reticular nucleus (Fig. 2). In the DRG, virtually all cells expressed Nedd4-2 and, in the spinal cord, a high level of expression was noted throughout the gray matter. Interestingly, the laminae I-III of the spinal cord, an area where DRG nociception unmyelinated afferent fibers project revealed the highest level of expression^16^; (Fig. 2G–I).

### Nedd4-2 heterozygous mice are hyperactive

**Figure 3:**
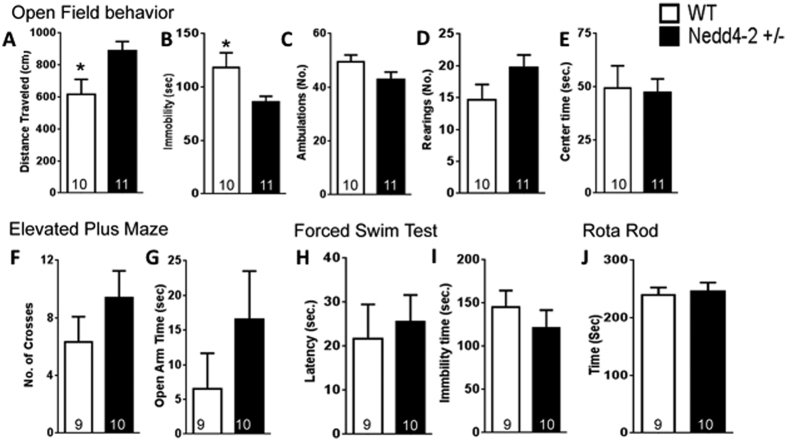
Nedd4-2 heterozygous mice have increased locomotor activity. Animals were subjected to the open field (**A–E**) elevated plus maze (**F,G**), forced swim (**H,I**) and rota-rod (**J**) test. Histograms showing the results of the general behavioral characterization of Nedd4-2 heterozygous mice reveal increased exploratory behavior in the open field test (**A–E**). No changes in anxiety (**F,G**), depression (**H,I**), or muscle strength/co-ordination (**J**) were observed. Numbers of animals per group are indicated inside the histogram. N = 9–11. Results are expressed as the Mean ± SEM. *Indicates p < 0.05; Mann-Whitney test.

Since Nedd4-2 is highly expressed in the brain, to further evaluate its function on general physical and behavioral characteristics we subjected heterozygous mice to a battery of behavioral tests aimed at evaluating general locomotor activity, anxiety and/or depression-related behavior as well as muscle strength and coordination. Interestingly, we found that in the open field test, the total distance traveled by Nedd4-2 +/− mice was significantly higher compared to that of WT mice (Fig. 3A). This result was also confirmed by a concomitant reduced immobility time recorded for the mutant mice (Fig. 3B). We then evaluated these mice in the elevated plus maze test for assessment of anxiety related behavior. Although we found a tendency to an increase in the number of crosses between different arms of the maze and the time spent in the open arm for the Nedd4-2 +/− mice these data were not significant (Fig. 3F,G) suggesting that the increased activity of the Nedd4-2 mice in the open field test is not related to anxiety-related behavior. This conclusion is further supported by the similar number of ambulations, rearing and time spent in the center of the open field recorded for both genotypes (Fig. 3C–E). In addition, data from the forced-swim and rota rod test revealed no difference in depression-like behavior and muscle strength/coordination between the two groups of mice (Fig. 3H–J). Nevertheless, the significant alterations found in the open field analysis suggest that changes in Nedd4-2 levels can influence behavior.

### Nedd4-2 heterozygosis increases the hippocampal response at low fEPSP stimulus intensity

**Figure 4:**
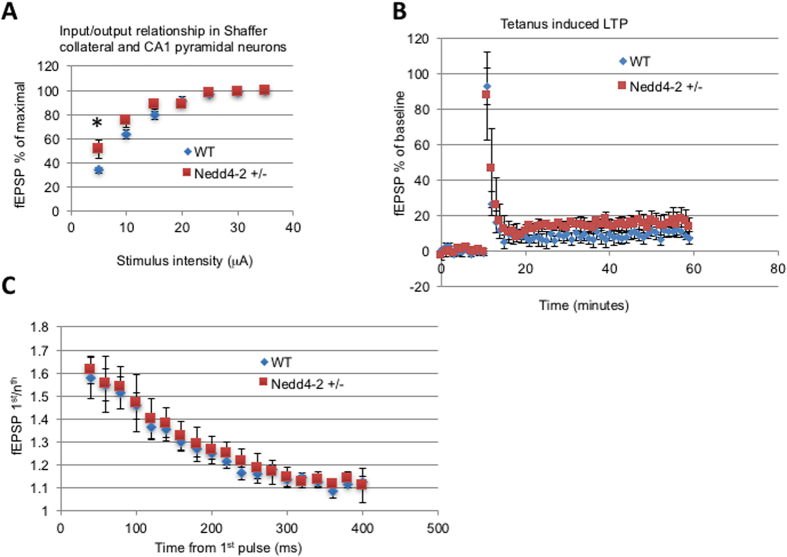
Nedd4-2 heterozygosis increases the response at low fEPSP stimulus intensity without affecting hippocampal basal synaptic transmission and Long-Term Potentiation (LTP). Extracellular recording in CA1 radiatum of field Excitatory Post Synaptic Potentials (fEPSP) obtained by stimulation of Schaffer collaterals. (**A**) Input/Output curve; initial slope of fEPSP obtained with increased stimulus intensity were plotted relative to the response normalized to the maximal response obtained (n = 18 per genotype). Note the statistically significant higher response at low stimulus intensity caused by NEDD4-2 reduction level. (**B**) Slope of fEPSP recorded in Wild-Type and NEDD4-2 +/− mice. Note that the LTP induced by high frequency stimulus is not statistically different between the 2 genotypes (n = 9 for each genotype). (**C**) Ratio of slope of paired fEPSP recorded at increasing interpulse time showing similar paired pulse facilitation between genotypes (n = 9 for each genotype).

To further characterize whether the Nedd4-2 haploinsufficiency may result in deficits that are not immediately apparent by behavioral or morphological characterization, we decided to analyze the basal synaptic transmission and LTP in these mice. LTP in the CA1 area of the dorsal hippocampus at 1 h after the conditioning in Nedd4-2+/− animals was similar to that found in WT mice, suggesting that loss of one copy of the gene does not affect this aspect of hippocampal synaptic plasticity (Fig. 4). However, Nedd4-2+/− mice had a significantly higher response at low stimulus intensity in the basal synaptic transmission, as assessed by the input–output curves (stimulus intensity vs. fEPSP slope) from the Schaffer collateral–CA1 region. These data suggest that in Nedd4-2 heterozygous mice, although LTP and presynaptic function are unaffected, there is a marginal increase in excitability of the Schaffer collateral -CA1 pathway (Fig. 4).

### Nedd4-2 heterozygote mice show increased sensitivity to inflammatory stimuli

**Figure 5:**
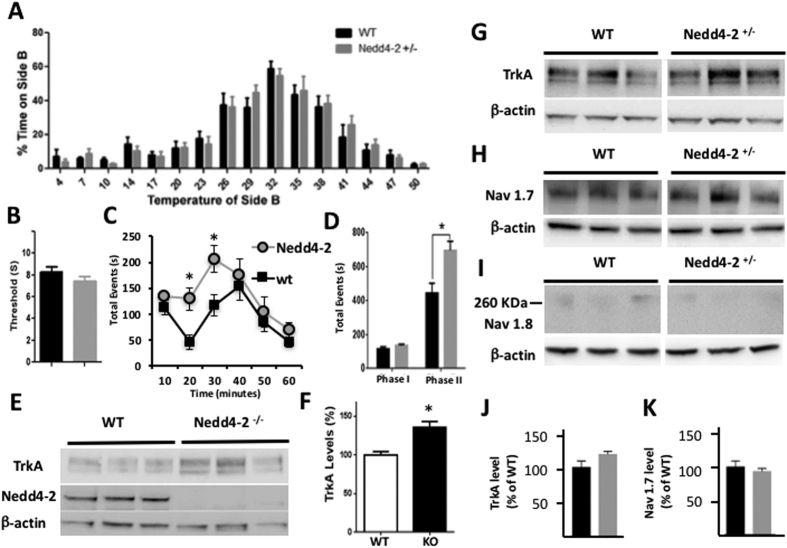
Nedd4-2 heterozygous mice have increased sensitivity to chemical stimuli. (**A**) In the two-temperature choice test there is no difference between mutants and controls in response to variable temperature. Side A of the chamber was kept at the constant temperature of 32 °C whereas side B of the chamber had variable temperature ranging from 4 to 50 °C as described in Methods. (**B**) Nedd4-2 heterozygous mice do not have an altered threshold in the Hargreaves test compared to controls. Graph showing the average time (in seconds) by which the mice responded to the thermal stimuli. (**C**) Nedd4-2 +/− mice have increased sensitivity in the early Phase II formalin test (two-way ANOVA, P < 0.05). Time course of the nocifensive response to formalin injection. n = 9–11 for each genotype in each test. *Indicates p < 0.05. (**D**) Quantification of total nocifensive responses from (**C)**. (**E,F**) Nedd4-2 loss increases TrkA expression levels in DRG. Western blot analysis of TrkA and Nedd4-2 protein in E13.5 mouse DRGs (**E**) and quantification of relative TrkA protein levels (**F**). TrkA band intensity was normalized relative to the intensity of the β-actin band used as control for loading control. Data are expressed as percentage of the mean TrkA levels in WT DRG. N = 3 sets of pooled DRG from different embryos per genotype *p = 0.0147, Error bars are S.E.M. (**G–K**) Western blot analysis of TrkA (**G**), Nav1.7 (**H**) and Nav1.8 (**I**) protein levels in lysates from +/+ and +/− adult mouse DRG. β-actin was used as a control for loading. (**J,K**) Quantification of TrkA (**J**) and Nav1.7 (**K**) band intensity as shown respectively in (**G**,**H**) (n = 3 sets of pooled DRG from different mice).

The hyperactivity of Nedd4-2 heterozygous mice and the higher response at low stimulus intensity in the basal synaptic transmission suggest that haploinsufficiency of this E3 ligase could have other consequences on nervous system function. Since Nedd4-2 deficiency in a conditional mouse model has been linked to neuropathic pain we investigated whether its partial systemic loss, which could mimic a human condition caused by the presence of loss of function SNPs, affects pain sensitivity^10^. In the two-temperature choice test, in which mice can choose to spend time on a plate at either 32 °C or a plate at different temperatures, we found that WT and Nedd4-2 heterozygous mice spent the same amount of time at either cold or hot temperatures (Fig. 5A) suggesting a similar sensitivity to thermal stimulation. This result was further confirmed in the Hargreaves test, that showed no difference in the latency threshold for flinching or paws withdrawal to a radiant heat stimulus in the two groups of mice (Fig. 5B) suggesting no changes in sensitivity to noxious thermal stimuli. However, when we measured spontaneous pain behavior in the formalin test we found that Nedd4−2 +/−mice were more sensitive than controls. In Phase I, corresponding to acute nociception immediately after the injection, there was no significant difference between genotypes. However, in the early Phase II, during the central sensitization and inflammation period mutant mice displayed a significant increase in licking and lifting/favoring of the injected paw as compared with their WT littermates suggesting a central consequence of increased peripheral activity due to loss of one Nedd4-2 allele (Fig. 5C,D). Taken together, these results indicate that Nedd4-2 heterozygous mice are normal in response to thermal stimuli but are hypersensitive to inflammatory stimulus. Next, we investigated the level of expression of some putative Nedd4-2 substrates that have been linked to this phenotype. Analysis of the sodium channel Nav1.7^10^ and Nav1.8 and the tyrosine kinase receptor TrkA^17^,^18^ in adult heterozygous DRG showed no change in their level of the expression (Fig. 5G–K). The lack of change in Nav1.7 was not surprising because its level was unchanged even in mice with a more drastic reduction of Nedd4-2 in DRG. However, Nav1.8 lack of up-regulation was more surprising because of the drastic increase observed in the Nedd4-2 conditional mutant mouse model^10^. These data suggest that most likely a combination of subtle changes in other sodium channels shown to be substrate of Nedd4-2^9^ may be involved in this phenotype. However, TrkA is the only Trk receptor targeted by this E3 ligase^19^. Indeed, analysis of DRG of Nedd4-2 −/− embryos showed a significant increase in the level of TrkA compared to WT controls confirming that TrkA is a direct Nedd4-2 substrate *in vivo* and there is no significant redundancy by other Nedd4 ligases (Fig. 5E,F). This result suggests that partial loss of Nedd4-2 in the adult may impact, although not significantly, the level of TrkA which could result in the increase in the pain sensitivity phenotype^20^.

## Discussion

Conditional gene deletion in mouse models is a powerful tool to investigate organ or cell-type specific functions of genes that are essential for viability. However, when a gene is haploinsufficient, heterozygous mice allow for the study of the significance of specific polymorphisms that potentially cause loss of function of one allele and that are associated to disease in human (e.g.^21^,^22^). Nedd4-2 ubiquitinates a variety of substrates including NaV channels expressed in lung, kidney and the nervous system^1^. Nedd4L polymorphisms have been linked to hypertension in human and, in mouse, Nedd4-2 heterozygosis leads to elevated baseline blood pressure compared to controls^3^,^23^. These observations support the notion that the Nedd4-2 heterozygous mouse may be a valid model to study the biological significance of SNPs of this E3 ubiquitin ligase^7^,^23^,^24^. We have found that a reduced level of Nedd4-2 causes an increase in locomotor activity and sensitivity to inflammatory pain. Although LTP induced by high frequency stimulus is not affected in Nedd4-2+/− mice, there is a significant higher response at low stimulus intensity in the basal synaptic transmission suggesting that a full complement of this gene is required for normal function of the nervous system. Importantly, it is possible that a higher response at low stimuli in the Nedd4-2+/− hippocampi may lead to increased neuronal excitability that in humans has been linked to epilepsy^25^. Indeed, genetic evidence suggests that mono-allelic NEDD4L loss of function is associated with epileptic encephalopathy^13^. However, it is unclear whether other genetic mutations are required for this phenotype since only one case was reported in that study^13^. Although a report has described missense mutations in highly conserved residues of NEDD4L in families with photosensitive generalized epilepsy, another study has failed to link NEDD4L variants to photosensitive epilepsy in a different population^26^,^27^. A mouse model with a severe Nedd4-2 loss of function has shown an increased susceptibility to epilepsy suggesting that while Nedd4-2 is important for this pathology, loss of one allele may not be sufficient unless other genetic mutations are present^28^. Introducing other candidate gene mutations onto the Nedd4-2+/− genetic background may provide a useful model to elucidate the genetics of this complex disorder^13^.

Recently, the results of the Epilepsy Comorbidity and Health (EPIC) survey have shown that epilepsy is often associated to pain manifestations including migraine headache, chronic pain, fibromyalgia or neuropathic pain^29^. Moreover, the finding that relatives of individuals with benign rolandic epilepsy have an increased frequency of migraine has suggested that migraine and some forms of epilepsy may have a genetic component^30^. Although our finding in the formalin test suggest that loss of one allele of Nedd4-2 leads to activity-related central sensitization and does not test chronic inflammatory pain it will be of interest to investigate whether these families have SNPs in the NEDD4-2 gene or a gene regulating its level of expression.

NEDD4-2 heterozygous mice are hyperactive, as judged by the increased locomotor activity and reduced immobility time in the open field test. NEDD4L SNPs have been associated to dyslexia^31^,^32^, a developmental reading disorder that is frequently comorbid with attention deficit hyperactivity disorder (ADHD)^33^. While it is difficult to associate dyslexia or ADHD to hyperactivity in mice it is tempting to speculate that these species-specific phenotypes may be caused by deficits in neuronal circuitries similarly affected by Nedd4-2 haploinsufficiency and Nedd4-2 heterozygous mice may serve as a model to study these human conditions.

A major finding of our study relates to the validation of TrkA as an important substrate of Nedd4-2. In fact, while *in vitro* and *in vivo* data have suggested that the TrkA binding site for Nedd4-2 regulates intracellular trafficking and function of this receptor in sensory neurons, it was unclear whether Nedd4-2 deficiency could directly impact TrkA level or be compensated by other Nedd4-like ligases^18^,^19^. Our data suggest that this receptor is a direct substrate of Nedd4-2 since Nedd4-2 developmental loss causes an increase of TrkA in DRG (Fig. 5E,F). Although we did not observe a significant TrkA upregulation in adult DRG from Nedd4-2−/+ mice it is possible that a reduction in Nedd4-2 may affect the subcellular distribution or responsiveness of TrkA to NGF^34^. Indeed, we have shown that a specific three amino acid domain in the TrkA juxtamembrane region that impairs TrkA ubiquitination leads to an increase in TrkA responsiveness to NGF which most likely contributes to the increased sensitivity to inflammatory stimuli^20^. Nevertheless, it is unlikely that TrkA is the only substrate responsible for the increase in the inflammatory pain, and a more plausible scenario includes the dysregulation of other substrates^10^. For example, there are at least three voltage-gated sodium channels (Na_V_1.7, Na_V_1.8, and Na_V_1.9) that are expressed in the DRG and the combined small changes of some of them may contribute to the overall increase in neuronal excitability despite the lack of obvious biochemical changes^35^.

In summary, we have found that mice with reduced levels of Nedd4-2 have behavioral and electrophysiological deficits that suggest that a full complement of this E3 ubiquitin ligase is necessary for the homeostasis and/or function of the nervous system. These data are in agreement with findings in cortical neurons where Nedd4-2 has been shown to mediate activation-induced down regulation of NaV that control neuronal excitability^8^. The significant increase in inflammatory pain points to Nedd4-2 as a major player contributing to the pathophysiology of pain. Most importantly, our model suggests that individuals with SNP in the Nedd4-2 gene may be susceptible to neuropathic pain and families affected by this disease should be genotyped for mutations of this gene.

## Materials and Methods

### Generation of Nedd4-2 mutant mice

Nedd4-2 mutant mice were generated from a gene-trap Nedd4-2 embryonic stem cell line obtained with the pGTOlxf vector (Baygenomics database, cell line ID: CA0066) with a strategy similar to that used in Puverel *et al.*^36^. By RT-PCR, we confirmed that the βGeo-containing gene-trap cassette was inserted in the intron following the 10th *Nedd4-2* coding exon. Mutant ES cells were injected into C57BL/6^CR^ blastocysts by standard methods to generate chimeric mice that transmitted the mutated allele to their offspring^37^. *Nedd4-2*^+/−^ mice were backcrossed to the C57BL/6^CR^ background for at least 6 generations before use. Mice group-housed under standard conditions with food and water available *ad libitum* were maintained on a 12 hr light/dark cycle and fed a standard chow diet containing 6% crude fat. All experiments were performed in compliance with the National Institutes of Health guidelines for animal care and use of experimental animals and all animal protocols were approved by the Animal Care and Use Committee of the National Cancer Institute, Frederick, MD.

### β-Galactosidase staining

After perfusion-fixation of the animals, brains and lumbar spinal cords were collected and post-fixed in 4% PFA for 2 h and then cryoprotected overnight in PBS containing 30% sucrose. Cryostat sections (12 μm thick for DRGs, 50 μm for brain and spinal cord) were mounted on Superfrost Plus slides (for DRGs) or collected in 1XPBS in 12 well plates (for brain and spinal cord). Free floating sections (brain and spinal cord) or slides (DRGs) were stained by incubation for two hours at 32 °C in a 4 mg/ml X-Gal (Sigma), 5 mM potassium ferricyanide, 2 mM MgCl_2_ and 0.25% Triton X-100 solution in PBS.

### Behavioral Analysis

All behavioral analysis was performed with male mice to avoid variations due to gender. *Open field test.* For the assessment of general locomotor and open field activity, animals were evaluated in an open field apparatus (Omnitech Electronics Digiscan animal activity monitor). Each mouse was placed in the arena (a 16 × 16 inch plexiglas chamber) for 5 min. Animal movements including locomotor activity (total distance traveled), freezing behavior (immobility), rearings, ambulations, and time spent in the center part of the field were recorded by infrared sensors mounted on to the walls of the chamber.

#### Elevated Plus Maze

Assessment of anxiety related behavior was done using an Elevated Plus Maze Apparatus (Biobserve, Germany) as described^38^. Briefly, mice were placed in the center of a plus maze with two open arms and two closed arms. Exploratory activity was monitored for 10 min with a video tracking system and the number crosses between the arms and time spent in the open arm was calculated for each animal.

#### Forced Swim test

To detect possible depressive behavior, animals were tested in the Porsolt swim test in a plexiglass cylinder as described^38^. Briefly, animals were placed in the water and swimming behavior was recorded with a video camera for 6 min. The video recordings were scored by an observer blind to the genotype. The latency to immobility and total immobility time during the last 4 min were calculated for each animal.

#### Rota rod test

Evaluation of muscle strength and coordination of mice was performed with a rota rod apparatus (Ugo Basile) as previously described^39^. Briefly, animals were placed on a rotating rod accelerating from 5 to 40 rpm over a 5 min period and the time spent on the rotating rod was averaged over three trials separated by 1 h intervals.

#### The Hargreaves test

*M*ice were placed in individual plexiglas chambers set on a glass plate (IITC Life Science apparatus) maintained at 30 °C and allowed to acclimate for 1 h. Once acclimated, the response latencies (flinching or paw lifting) to noxious thermal stimulation were measured by applying a radiant heat stimulus to the glabrous skin of each hindpaw. Three measurements from the left paws were averaged to obtain a single score for each animal^20^.

#### Formalin test

was performed as previously described^20^. Briefly, animals were placed in Plexiglas boxes on top of a glass plate to be acclimated for 30 min in an isolated behavioral room before formalin injection. For the test, animals were anesthetized briefly by isoflurane vapor (using a nose cone) and subcutaneously injected with 25 µL of 1.85% formaldehyde in 0.9% saline to the right hindpaw. Then, they were immediately replaced into the Plexiglas chamber and video recorded for 60 min. The total amount of licking/biting of the affected hind paw (counted as number of events) was measured in 10 min intervals in a blind fashion. The acute phase (Phase I) is defined as 0–10 min after injection, and the persistent (tonic) phase (Phase II) is defined as 10–60 min after injection.

#### Two-temperature choice test

Experimental mice were placed into a thermal gradient apparatus (IITC Life Science) and allowed to explore two adjacent areas. One was held at a constant 32 °C (side A) while the other side had variable temperatures ranging from 4 to 50 °C (side B). Mice were placed in the apparatus for 10 min, and the time spent in each area was recorded. The percentage of time spent on side B during the last 5 min interval was measured^20^.

### Western blot analysis

DRG neurons were isolated from E13.5 embryos or adult animals. After homogenization with 1× RIPA lysis buffer (Millipore, USA), supernatants were mixed with Laemmli’s sample buffer (Sigma, USA), boiled at 95 °C for 5 min, subjected to electrophoresis and transferred to PVDF membranes by electroblotting. After blocking with 5% milk in TBST, membranes were incubated with Rabbit anti-TrkA (1:1000 Advanced Targeting Systems), Mouse monoclonal anti-NaV1.7 (1:500, N68/6 NeuroMab Facility, UC Davis); anti-NaV1.8 (1:200, NeuroMab); Rabbit anti-Nedd4-2 (1:1000, Abcam) or b-actin (1:1000, Santa Cruz Biotechnology) specific antibody overnight. After washing and incubation with HRP conjugated secondary antibody, the membranes were imaged with a GeneGnome Imager with Gene Snap software (Syngene Bio-imaging system, USA) using ECL detecting reagent. Band intensity was quantified using GeneTools Software (Syngene Bio-imaging).

### Electrophysiological studies

Adult mouse brain slices were prepared as described^40^. Briefly, after isofluorane anesthesia, mice were transcardially perfused with 20 ml of NMDG solution (92 mM N-methyl-D-glucamine, 2.5 mM KCl, 1.25 mM, NaH_2_ PO_4_, 30 mM NaHCO_3_, 20 mM HEPES, 25 mM glucose, 2 mM thiourea, 5 mM Na-ascorbate, 3 mM Na-pyruvate, 0.5 mM CaCl_2_, and 10 mM MgSO_4_, titrated to pH to 7.3–7.4 with HCl) and the brains were rapidly removed and sectioned coronally (300 µm thick sections) with a Leica (VT1200) vibratome. Slices were incubated for 10 minutes in NMDG solution and then placed in a holding solution (92 mM NaCl, 2.5 mM KCl, 1.25 mM NaH_2_PO_4_. 30 mM NaHCO_3_. 20 mM HEPES, 25 mM glucose, 2 mM thiourea, 5 mM Na-ascorbate, 3 mM Na-pyruvate, 2 mM CaCl_2_, and 2 mM MgSO_4_) in a chamber oxygenated with 95% O_2_ and 5% CO_2_ for at least 1 hour. For recording, a slice was placed in a recording chamber and perfused with ACSF (119 mM NaCl, 2.5 mM KCl, 1.25 mM NaH_2_PO_4_. 24 mM NaHCO_3_. 12.5 mM glucose, 2 mM CaCl_2_, and 2 mM MgSO_4_) at the rate of 2 ml/min. A pulled recording borosilicate glass electrode (2–3 MOhm when filled with ACSF) was placed in the CA1 radiatum and two stimulating electrodes with a concentric tungsten tip were placed on both side of the recording electrode in the Schaffer collateral. Field EPSP (fEPSP) was obtained by alternating stimuli applied every 10 seconds at the 2 electrodes. Stimulus (100 µS long) intensity was adjusted such that the 2 electrodes would elicit approximately the same response. We avoided LTP saturation to facilitate the detection of possible differences between genotypes, by employing a mild LTP protocol consisting of two, 250 mS long, 100 Hz trains, 20 seconds apart induced by the electrode close to the CA3 while the second electrode was used to control the status of the slice. Field potential was recorded (Multiclamp 700 b; Molecular Devices), digitized (10 kHz Digidata 1324), low-pass filtered (3 kHz, eight-pole Bessel), and stored (Clampex 9.2; Molecular Devices). Signals were analyzed offline (Clampfit 9.2; Molecular Devices), and the size of the fEPSP was evaluated by measuring the initial slope of the signal expressed as percentage of the variation from the baseline value (average of 5 min before the conditioning protocol). Results were further analyzed with Igor Pro 6.01 (WaveMetrics). All data are reported as means ± SEM.

### Statistical Analysis

Data were analyzed using the Statistical Software (GraphPad Prism, USA). For comparisons between the groups, the group means were subjected to two-tailed Student’s *t* test or non-parametric Mann Whitney test.

## Additional Information

**How to cite this article**: Yanpallewar, S. *et al.* Nedd4-2 haploinsufficiency causes hyperactivity and increased sensitivity to inflammatory stimuli. *Sci. Rep.* **6**, 32957; doi: 10.1038/srep32957 (2016).
